# Factors Affecting Clinician Readiness to Adopt Smart Home Technology for Remote Health Monitoring: Systematic Review

**DOI:** 10.2196/64367

**Published:** 2024-12-05

**Authors:** Gordana Dermody, Daniel Wadsworth, Melissa Dunham, Courtney Glass, Roschelle Fritz

**Affiliations:** 1 School of Health, Nursing University of the Sunshine Coast Sippy Downs Australia; 2 Manna Institute University of New England Armidale Australia; 3 School of Nursing & Midwifery Edith Cowan University Joondalup Australia; 4 The Betty Irene Moore School of Nursing University of California Davis, CA United States

**Keywords:** clinician, provider, health professional, smart home, remote monitoring, technology, readiness, adoption, preparedness

## Abstract

**Background:**

The population of older adults worldwide continues to increase, placing higher demands on primary health care and long-term care. The costs of housing older people in care facilities have economic and societal impacts that are unsustainable without innovative solutions. Many older people wish to remain independent in their homes and age in place. Assistive technology such as health-assistive smart homes with clinician monitoring could be a widely adopted alternative to aged-care facilities in the future. While studies have found that older persons have demonstrated a readiness to adopt health-assistive smart homes, little is known about clinician readiness to adopt this technology to support older adults to age as independently as possible.

**Objective:**

The purpose of this systematic review was to identify the factors that affect clinician readiness to adopt smart home technology for remote health monitoring.

**Methods:**

This review was conducted in accordance with the Joanna Briggs Institute methodology for systematic Reviews and followed the PRISMA (Preferred Reporting Items for Systematic Reviews and Meta-Analyses) guidelines for reporting.

**Results:**

Several factors affected clinicians’ perspectives on their readiness to adopt smart home technology for remote health monitoring, including challenges such as patient privacy and dignity, data security, and ethical use of “invasive” technologies. Perceived benefits included enhancing the quality of care and outcomes.

**Conclusions:**

Clinicians, including nurses, reported both challenges and benefits of adopting smart home technology for remote health monitoring. Clear strategies and frameworks to allay fears and overcome professional concerns and misconceptions form key parts of the Readiness for Adoption Pathway proposed. The use of more rigorous scientific methods and reporting is needed to advance the state of the science.

**Trial Registration:**

PROSPERO International Prospective Register of Systematic Reviews CRD42020195989; https://www.crd.york.ac.uk/prospero/display_record.php?RecordID=195989

## Introduction

### Background

The global population is aging, and people are living longer. Adults aged ≥65 years have a high burden of disease, and the current practice of housing and caring for older people in nursing homes is often considered the last resort [1]. With reports of lower-quality care in residential aged-care settings and demands for alternative aging-in-place solutions by a much more technology-savvy baby boomer generation [2,3], health-smart homes (HSHs) are capable of unobtrusive in-home monitoring to support the health and safety of older people. In addition to protecting older adults from unnecessary exposure to communicable diseases such as COVID-19, aging in place has several benefits, including the maintenance of social connectivity and proximity to friends and family [1,4,5]. Furthermore, the ability to age in place supports and maximizes independence, thereby enhancing well-being and quality of life while decreasing the financial burden of residential care costs [1,6,7]. Older adults are a unique population with age-related changes and conditions and yet could remain living in their ancestral homes with the help of HASs, which could also augment public or private home care services. However, while HAS technologies are maturing, to improve wider use of the HAS, gaining an understanding of clinician readiness to use HAS technologies is important because clinicians that make up the multidisciplinary care team constitute important stakeholders [8].

### Smart Home Technology for Remote Health Monitoring

The term Internet of Things refers to a collection of “smart” devices that can acquire and connect data or information across environments and act on the information [9], for example, by sending an alert that a person’s overall activity has significantly decreased. Although wearables can be used in an in-home context, there are many different types of wearables for different purposes and that collect different types of data compared with the health-assistive smart home. In addition, wearables usually do not attract some of the privacy concerns associated with remote monitoring using health-assistive smart home technologies. The health-assistive smart home consists of Internet of Things devices such as unobtrusive sensors that are deployed in a home to monitor a person’s routine behaviors and activities of daily living, including movement around the home such as sleeping, eating, steps ambulated, and more without the person having to “wear” a device [10]. The health-assistive smart home can collect and analyze a variety of data with the help of intelligent algorithms. Data can then be used by clinicians to monitor potential changes in health in their older patients [11,12]. Clinicians and the multidisciplinary care team are positioned to be the primary end users of patient data derived from the smart home sensors. Smart devices enable clinicians to unobtrusively monitor their older patients using automated assessment of behaviors that are associated with changes in health, which could support pragmatic, data-driven clinical decision-making [13].

### Readiness and Acceptance Models

Readiness and acceptance models have been developed to help integrate computer-based information systems and digital technologies into specific settings, including health care. Researchers have studied the factors that may impact acceptance of and readiness for new technologies for several decades [14,15]. Examples of studies that explore technology readiness and acceptability in clinicians include mobile electronic health records [16], information and communications technologies [17], electronic care plan systems [18], telemedicine readiness [19], and computer-generated nursing care plans [20]. Frameworks and theories have been developed to understand a person’s likelihood of accepting and using health technologies [21]. The technology acceptance model (TAM) is the predominant framework cited in the literature [22-24]. This framework is used to model the behavioral intention that leads people to accept a certain technology [22-24]. In this model, behavioral intention is influenced by the person’s attitude generated from their impression of the perceived usefulness of the technology, which then predicts the actual use of the technology [22-24]. The TAM was expanded to include social influences and cognitive instrumental processes (TAM 2) [22], and later, the TAM 2 was combined with determinants of perceived ease of use to form the TAM 3 [23]. Determinants of perceived ease of use included computer self-efficacy, perception of external control, computer anxiety, computer playfulness, perceived enjoyment, and objective usability [23]. The Unified Theory of Acceptance and Use of Technology was developed from previous work with the aim of explaining end-user intention to and use of IT [24]. Models can help with intentionally integrating computer-based information systems and digital technologies into specific settings, including health care. However, there are larger differences between adopting, for example, an electronic health record and the readiness of clinicians to adopt home health monitoring using smart home technology. Clinician adoption of smart home technology requires clinicians to use and understand a new form of evidence. Accordingly, using the findings of this systematic review, we developed a theoretical model to support clinician readiness for and adoption of HAS technology, which will be discussed at the end of the *Results* section.

### Clinician Readiness

The research and development surrounding the health-assistive smart home is maturing, with deployment as part of research studies across a variety of care settings, including private homes, assisted living, residential memory care, and residential care settings such as nursing homes [25-27]. Accordingly, the readiness of clinician end users to adopt smart home technology–generated data for clinical decision-making is important if wider adoption of the health-assistive smart home is desired. Clinicians such as nurses, physicians, and other allied health professionals will play a key role in monitoring older adults living using health-assistive smart home technology. As end users, clinicians will need to use sensor data integrated with other health information and apply clinical judgment to triage information and liaise with the multidisciplinary health care team for early interventions [28,29]. Exploring factors that may impact readiness to adopt, including perceived benefits and challenges, is needed to inform the integration of the HAS into new models of home care and clinical practice. The purpose of this systematic review was to identify and summarize the factors that may impact clinician readiness to adopt smart home technology for remote health monitoring.

## Methods

### Review Question

What are the factors affecting clinician readiness to adopt smart home technology for remote health monitoring of community-dwelling older adults?

### Inclusion Criteria

#### Participants

A preliminary search showed that, frequently, a variety of clinicians and other stakeholders have been included in studies that examined the phenomenon under review. Accordingly, clinicians aged ≥18 years, such as nurses, registered nurses, clinical nurses, physicians, allied health professionals, and other health care workers, were included. Studies that included nonclinical stakeholders as participants were included if clinicians were also included. Studies were excluded if we were unable to identify clinician participation.

#### Interventions

Because of the unique data that the health-assistive smart home can generate with the potential to augment clinician decision-making, this review included studies on health-assistive smart home sensor technology embedded or deployed in the home environment (eg, ceilings, walls, furniture, and appliances) to detect motion in persons living in private dwellings in the community, retirement villages, or aged-care homes and residential care homes. Studies focusing on telehealth and remote health monitoring were included if smart home sensors were also embedded in the home. Studies focusing only on wearable sensors or other technologies used for remote monitoring (eg, implanted defibrillators) were beyond the scope of this review and were excluded. Studies that did not provide sufficient information to ascertain whether smart home sensors were embedded or deployed in the home environment were also excluded.

#### Comparators

This review considered studies that compared the use of smart home technology to usual care. Studies that did not use comparisons such as cohort, case studies or descriptive qualitative studies, were also included. Due to the challenges with the feasibility of implementing the health-assistive smart home in real-world settings, studies that used “mock” or hypothetical health-assistive smart homes to study clinician readiness were included.

#### Outcomes

The primary outcome of this review was the identification of factors that may impact clinician readiness to adopt smart home technologies. Studies reporting clinician feedback, attitudes, perceptions, and experiences, including barriers, facilitators, and enablers regarding smart home technology, were included. Studies that reported nonclinical stakeholder feedback were included if clinicians were also included in the study.

#### Types of Studies

This review considered experimental and quasi-experimental studies, randomized controlled trials (RCTs), qualitative studies, case studies, and surveys. Human studies conducted in any geographical area and published in English from database inception to July 2024 were included.

### Review Registration

The review protocol was registered with PROSPERO (CRD42020195989) before the commencement of the database searches.

### Review Methodology

This review was conducted in accordance with the Joanna Briggs Institute (JBI) methodology for systematic reviews and followed the PRISMA (Preferred Reporting Items for Systematic Reviews and Meta-Analyses) guidelines for reporting.

### Search Strategy and Study Selection

The initial search [30] of 12 databases up to July 2024 resulted in 17,204 references. Additional records were identified (n=24) through manual searching, including searching systematic review reference lists. After duplicates were removed, of the 17,228 study titles, a total of 13,423 (77.91%) were assessed. After the title search was completed, a title screen was performed by MD, and abstract screening was performed independently by 4 reviewers (GD, CG, MD, and DW). Full-text screening was performed independently by GD, CG, MD, and DW. A total of 155 full-text articles were comprehensively assessed for inclusion. Disagreements arising between the reviewers were resolved through discussion until consensus was reached. Of the 155 full-text articles, a total of 128 (82.6%) were excluded for the reasons previously stated. A total of 27 papers addressing clinician readiness, perspectives, and attitudes, among other things, regarding smart home technology were included in this systematic review.

**Figure 1:**
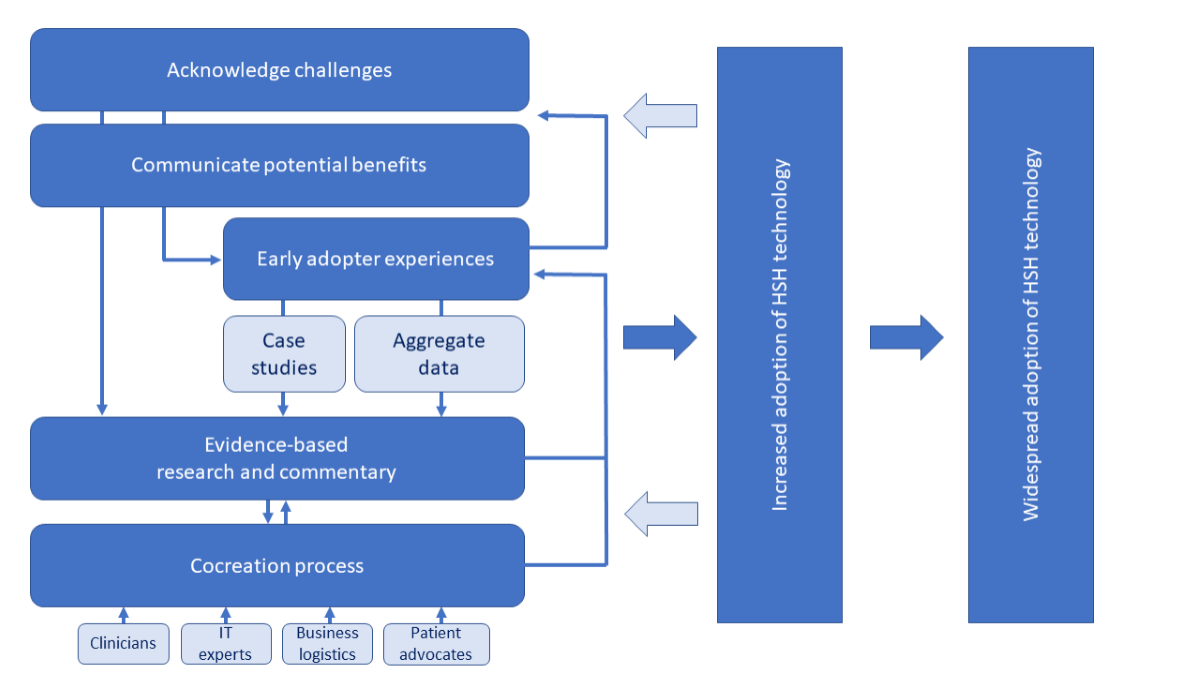
Model for clinician adoption of smart home technology. HSH: health-smart home.

The results will inform researchers and clinicians regarding the challenges and perceived benefits of adopting smart home technology for remote health monitoring. Finally, the findings of this review were used to develop a smart home adoption model for clinicians, which is presented in Figure 1.

### Assessment of Methodological Quality and Certainty of Findings

Eligible studies were critically appraised, and methodological quality was assessed by independent reviewers (GD, CG, MD, and DW) using the standardized critical appraisal instruments from the JBI [31]. This allowed the reviewers to achieve a greater insight into the methodological strengths and limitations of the selected studies. Blinding treatment groups was not always conceivable given the nature of the intervention; hence, it was not considered a criterion for inclusion. Any incongruities in appraisal that arose between reviewers were discussed and resolved by all authors. The Grading of Recommendations, Assessment, Development, and Evaluation (GRADE) approach for assessing the certainty of evidence for an effect, summarized in narrative form, was used to assess the overall quality of the findings of each paper [31]. The GRADE assessment evaluates the limitations, indirectness, imprecision, inconsistency, and publication bias of the studies [31]. The overall quality of the evidence was then categorized as high, moderate, low, or very low. In total, 2 reviewers (GD and DW) independently completed the GRADE assessment for each article; there were no disagreements.

### Data Extraction

Data were extracted from the studies by 2 independent reviewers (GD and DW) using an adapted version of the JBI standardized data extraction tool. Extracted data included specific details about the populations, study methods, types of smart home technology intervention used, outcomes assessed, and themes relevant to the review objective. Disagreements arising between the reviewers were resolved through team discussions. Both team members’ input was equally valued and used when coming to agreements.

### Data Synthesis

As the studies included were heterogeneous, the findings of the selected studies were narratively synthesized to examine the barriers, facilitators, enablers, perceptions, and attitudes of clinicians and how these factors may impact the readiness to adopt smart home technology.

## Results

### Summary of the Search Results

**Figure 2:**
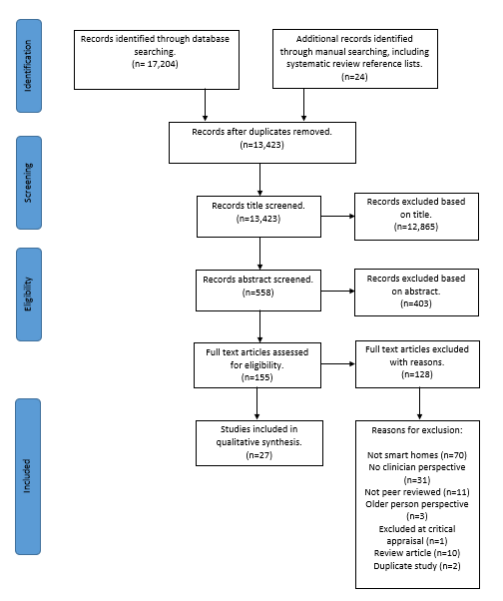
PRISMA (Preferred Reporting Items for Systematic Reviews and Meta-Analyses) flowchart.

The initial search (Figure 2) of 12 databases resulted in 17,204 references. Additional records were identified (n=24) through manual searching, including searching of systematic review reference lists.

Table 1 presents the comparative characteristics of the studies and the GRADE rating. Table 2 presents the summary of the key factors that may affect clinician readiness to adopt smart home technology, which have been categorized into perceived challenges and perceived benefits. Multimedia Appendix 1 presents descriptive themes and subthemes. The results will inform researchers and clinicians regarding the challenges and perceived benefits of adopting smart home technology for remote health monitoring. Finally, the findings of this review were used to develop a smart home adoption model for clinicians, which is presented in Figure 1.

**Table 1:** Literature review table.

Study and country	Study design and purpose	Sample size and characteristics	Smart home technology	Outcomes assessed	Measures (GRADE^a^)
Beaudin et al [32], United States	Qualitative; to elicit specific feedback from health professionals and lay people on how they might use longitudinal health monitoring data for proactive health and well-being	N=34; 8 health professionals; 26 lay people; professionals in aging and cognition, geriatric nurses, home nurses, cognitive psychologists, and OTs^b^	Developed mock sensor visual displays representing a hypothetical patient in a variety of constructs used to elicit feedback on longitudinal tracking ideas	Reactions to longitudinal monitoring in the ho me; types of behaviors, events, and physiological indicators that participants would be interested in tracking; primary question: can monitoring systems be designed that might be adopted by end consumers for personal use?	Interviews (low)
Brand et al [33], United States	Descriptive; to ask multiple stakeholders to rate their level of concern about the privacy of individuals with disabilities when receiving services via smart homes	N=209; 16 volunteers; 44 direct service professionals; 37 administrators or coordinators; 22 managers; 2 licensed practical nurses; 38 licensed practitioners (eg, psychologists, social workers, and behavior analysts); 18 family members or self; 6 teachers, advocates, or attorneys; 3 counselors-job- coach-trainers; 7 participants with multiple roles listed; 16 not clearly identified or specified; 9.6% aged ≥65 years; 27.3% aged 55-64 years; 25.4% aged 45-54 years; 16.3% aged 35-44 years; 19.1% aged25-34 years; 2.4% aged18-24 years	Participants were shown a short explanatory video that described how smart home technology was used to provide services to individuals with disabilities using video cameras, motion sensors, and remote coaches	Information about levels of concern associated with individual smart home technology; privacy concerns across various aspects of smart homes	Survey (low)
Caprani et al [34], Ireland	Qualitative; to explore HCPs’^c^ preferences for using and visualizing sensor data	N=14; phase 1: 9 PTs^d^; phase 2: 0; phase 3: 2 OTs, 2 PTs, and 1 nurse; employment settings: community, nursing home, and community	Developed a mock system design based on clinician feedback consisting of smart home sensors to measure activity, gait, health, and sleep	How sensor technology can support the role of HCPs, technicians, and patients	Interviews (low)
Ding et al [35], United States	Qualitative; to explore the service delivery practices of mainstream smart home technology as assistive technology	N=15; 7 OTs, 6 assistive technology professionals, 4 aging-in-place specialists, 2 speech and language pathologists, 2 certified environmental access consultants, 1 PT, and 1 certified living administrator	Home automation devices to support independence in people with disabilities or older adults	Service delivery practice challenges and perspectives on benefits, limitations, and barriers	Interviews (low)
Cohen et al [36], Switzerland	Descriptive and qualitative; to explore the perception of acceptability among CHNs^e^ of an IWSS^f^ for use in daily practice for the detection of health issues in home-dwelling older adults receiving home care; secondary analysis of qualitative data from a pilot RCT^g^ of IWSS acceptability among home-dwelling older adults	N=17; CHNs were 88% (n=15) female; mean age of 26.4 years; practicing in home care for an average of 5.1 years	IWSS placed in living room, bedroom (time in bed), and refrigerator (tracked refrigerator door opening and closing). An algorithm analyzed and detected changes in behavior patterns regarding refrigerator opening and time in bed. CHNs received alerts via SMS text message followed by email and smart home reminders. The CHN had access to a smart application dashboard to discover the nature of the change in movements or activity patterns.	Perceived usefulness and perceived ease of use of IWSS among CHNs; number of alerts transmitted; relevance of IWSS alerts for CHNs in their daily practice	Survey and focus groups (low)
Delbreil and Zvobgo [37], Switzerland and the United Kingdom	Mixed methods; to examine the health professionals’ recognition of sensor technology to enhance the QoL^h^ of care recipients with dementia	N=60; comprised RNs^i^, medical assistants, PTs, OTs, and social workers; physicians were excluded	WSN^j^ technology	Effect of wireless sensor technologies on QoL; confidence of caregivers that wireless sensor technologies will lighten caregiver burden while satisfying end-user needs and QoL aspects; barriers to wireless sensor technology use; value derived in the older adult care sector from using sensor technology	Web-based survey (low-moderate)
Dugstad et al [38], Norway (based on the work by Nilsen et al [39])	Longitudinal case study; to identify facilitators and barriers and explore co-creation practices as an innovation strategy during the implementation of digital monitoring technology in LTC^k^ for persons with dementia who were night wanderers	Workshops: n=172 (89 municipal health care service staff members, 8 IT staff members, 30 vendors, 14 research institutions, 3 NGOs^l^, 5 other public-sector organizations, 20 innovation and funding organizations, and 3 external experts); interviews: n=16 (13 RNs and 3 HCWs^m^); focus groups:9 HCPs and 4 vendors	On the basis of the Digital Night Surveillance Innovation Project; using monitoring technology for a night surveillance intervention for increasing the safety of PLWD^n^ who wander; 67 installations of the monitoring technology were implemented; sensors included door sensors, electronic security blankets on mattresses to monitor bed exit, and SMS text message–mediated alarm	Facilitators of and barriers to monitoring technology in long-term residential aged care; co-creation practices as an innovation strategy	Workshops, interviews, and focus groups (low)
Elavsky et al [40], Czech Republic	Mixed methods; to explore the barriers and concerns related to the adoption of smart technologies among different groups of stakeholders	N=390; qualitative data:12 professional caregivers and 9 experts in aging; cross-sectional survey: 369 older adults attending the University of the Third Age	Smart home technologies in care for older adults; ANUME smart bed system	Barriers to the adoption of smart technologies in the care of older adults; solutions to overcome these barriers	Mixed methods (moderate)
Glasby et al [41], United Kingdom	Qualitative; evaluation of decision-making process and implementation of home-based sensor technology “IndependencePlus”	N=23; 3 decision makers and operational leads; 5 care staff members, care providers, and unpaid carers; 2 technology providers; and 1 regulatory organization	Home-based sensor technology “IndependencePlus” in 3 case study sites (1 care provider and 1 local authority); 1 care home with nursing services (23 clients) and 1 community care setting (9 clients); local authority with urban and rural areas (20-30 clients)	Decision-making process and implementation of “IndependencePlus”; barriers to and facilitators of the use of AI^o^ in social care	—^p^
Hall et al [42], United Kingdom	Qualitative; to explore the extent of the ethical considerations of implementing monitoring technologies in 3 dementia specialist care homes	N=24; aged 21-64 years, mean age 39.75 years; managers, clinicians, and support workers (specific numbers for each profession not provided)	Bed sensors, pressure mats, sensors in ceiling, door sensors, RFID^q^ location-based system, and accelerometer	Ethical considerations when using monitoring technologies in care homes; equality of access to monitoring technologies	Embedded multiple–case study design; interviews (low)
Hunter et al [43] (research brief) and Hunter et al [44]) original paper, New Zealand	Qualitative; to understand the perspectives of HCWs on the use of technology to provide aging in place to inform the development of a prototype system being tested in older persons’ homes	N=44; community-based care:5 nurses, 11 social workers, 3 PTs, 2 OTs, and 1 clinical coordinator; primary care practice:3 GPs^r^, 8 nurses, 3 directors, 1 business manager, 2 clerks, 2 geriatricians, and 1 social worker; predominantly female, with a mean age of 46.6 years	Smart home technologies and home monitoring devices, including technologies that involve interaction among societies, complex infrastructures, and human behavior	Health providers’ perspectives on the use of smart home technologies to provide services to older adults; types of information needs; the type of information that should be collected and transferred; the purpose of the information collected; who should receive the information; ethical concerns	Focus groups (low)
Ienca et al [45], Switzerland, Germany, and Italy	Qualitative; to explore the views and attitudes of health professionals and researchers involved in psychogeriatric care and research on IATs^s^ for dementia and older adult care	N=17; 41% female; professionals from gerontology, geriatrics, general practice, neurology, neuropsychology, nursing, nursing home management, and psychiatry	IATs discussed in interviews: distributed systems, robots, mobility and rehabilitation aids, handheld or multimedia, software apps, wearables, and human-machine interfaces	Health professional expectations, needs, and perceptions regarding IATs; practical experience with IATs; perceived effectiveness of IATs; health professional recommendations for improving IAT use for end users	Interviews (low)
Islam et al [46], Australia	Qualitative	N=9; clinicians from different settings involved in the clinical care of people with cardiovascular disease or heart failure; 3 cardiologists, 1 pharmacist, 4 nurses, and 1 GP	Smart home system for heart failure, including sensor-enabled medication monitoring; motion sensors; blood pressure, weight, and fat percentage monitoring; voice activation; wearable for activity; sleep and heart rate monitoring; and educational content, alerts, and messaging	Key HCPs’ perspectives on using smart home system to support self-management and home-based care in people with heart failure; types of technologies useful to support the self-management of patients with heart failure; barriers to and facilitators of uptake	Interviews (low)
Klemets et al [47], Finland	Qualitative; to determine how wireless sensor–collected information about an older adult’s physical motion can be used by nurses	N=9; nurses (5 from home care team)	Smart home project–developed prototype wireless infrared sensors placed in each room; monitors in-home physical activity, bathroom visits, physical motion, and time spent in different rooms and outside; data are presented as daily physical motion patterns	Challenges to nurses’ existing care provision and whether elementary information about older adults’ motion patterns could support their work	Interviews (low)
Klemets et al [48], Finland	Qualitative analysis of a case study; to determine how an in-home monitoring system can be used and integrated into home care nurses’ workflows and identify the factors that influence system adoption	N=4; 3 RNs and1 head nurse	IOT^t^ architecture with PIR^u^ motion sensors to monitor activities of daily living, including bathroom visits and time spent in rooms; sensors were placed in 8 residents’ apartments, and nurses captured their thoughts and reflections on the in-home monitoring system in a notebook; they were interviewed on the system’s use and usefulness; nurses used the in-home monitoring system 2 times weekly for 4 weeks	Nurses’ perspectives on a deployed in-home monitoring system	Focus groups and interviews (low)
Lee and Dey [49], United States	Case study (interviews); to determine how reflecting on sensor data about everyday activities will aid patients and their clinicians in making better informed decisions about their care	N=9; 6 primary care physicians and 1 oncology specialist; case studies of 2 older women (aged 81 and 77 years)	Ubiquitous sensor “dwellSense” system deployed in older adults’ homes for 10 months to monitor ODLs^v^ using smart pill box, phone sensor, and sensor-augmented coffee maker; relies less on machine learning and more on simple task-based sensors and heuristics applied to common tasks	Patients: support self-reflection, self-awareness, and improvement of their ability to live independently in their home; clinicians: how can clinicians make care plans based on information about patients’ ODLs based on data captured by the sensing system	Interviews (low)
Mahoney et al [50], United States	Multiphase mixed methods; to gain an understanding of the needs of independently living residents and whether remote residential monitoring using off-the-shelf wireless sensors might address these concerns	Predevelopment focus groups: n=26 (13 residents: mean age 7 9 years, widowed, and 66% female;4 family members [no details given]; 9 staff members: most were middle aged and female); intervention focus groups: n=29—10 residents, 10 family members, and 9 staff members (residents:60% female, White, mean age 83, range 70-91 years, and most were widowed [n=7]; family members: 60% female, White, mean age 56, range 40-76 years, and married [n=8])	AT EASE^w^ remote home monitoring system consisting of motion sensors in each room, water sensors in the bathroom, ability to remotely enable or disable the system, a processing unit with internet connection, and a Zigbee computer interface and custom automation software application; additional sensors available but not desired by participants: contact sensors for doors, pressure sensors for beds and chairs, and appliance on and off sensors	Types of concerns for staff, residents, and families for monitoring independently living residents; potential signal interferences due to density; monitoring system adaptability to the concerns of end users without security breaches and invalid alert notices	Focus groups and questionnaire (low)
Martin et al [51], Ireland and the United Kingdom	Qualitative; to investigate care staff perspectives on an ambient-automated home environment in dementia-specific housing to design a user interface	N=7; 5 care staff members and2 senior management stakeholders employed at the housing scheme	Discrete sensor network to support tenant activity and daily living tasks with minimum staff intrusion; sensors included PIR sensors, door contacts, pressure pads, and water and cooker valves	Staff perspectives on tenant activity had the potential to inform the care process; how this information should be presented on a computer user interface from the data monitor in the staff office	Interviews (low)
Newton et al [52], United Kingdom	Qualitative; to explore the views and experiences of people with dementia and their family carers and GPs regarding accessing information about and use of assistive technologies in dementia care	N=56; 17 GPs (mean age 42 years, including 6 trainees and 5 GPs with a commissioning role [mean age 30 years]), 13 people with dementia (mean age 72 years), and 26 family carers (mean age 61 years)	Photographic images including the following assistive technologies: community alarms and telecare, GPS location monitoring devices, signage, reminiscence tools, clocks to aid orientation, simplified telephones with pictures, and dementia-friendly furniture; personal and practical experience with assistive technologies, such as pendant alarms, fall alarms, door exit sensors, pill dispensers, signage, and easy-to-use telephones	Knowledge and experience of accessing information about and use of assistive technologies in dementia care	Interviews (low)
Nilsen et al [39], Norway	Longitudinal single-embedded case study; to identify and describe forms of resistance to the implementation of night surveillance technology in nursing homes and home care services	N=50 participants with 17 HCPs; network of small to medium-sized technology enterprises and3 municipal health and care services; university research group	Digital night surveillance system with digital communication, sensors on doors, and electronic security blankets on mattresses used during the night	Types of resistance among night care staff to the implementation of the monitoring technologies	Co-creation and implementation process using interviews, focus groups, observations in meetings, and workshops (low)
Offermann-van Heek and Ziefle [53], Germany	Mixed methods; to investigate the PC^x^ acceptance of assistive technologies in professional care contexts	N=170; 74.7% female (mean age 36.26, SD 11.23 years); 25.3% working in geriatric care, 22.9% working in medical care, and 51.8% working in care of people with disabilities	Scenario of integration of AAL^y^ system, including room sensors, microphones, video cameras, and ultrasonic sensors	PC acceptance of assistive technologies in 3 care contexts: geriatric, medical, and care and support of people with disabilities; PCs’ perceptions in different care contexts on AAL technologies, willingness to share care-related data, willingness to be assisted by specific AAL technologies in their daily routines, and which are the main predictor variables for AAL acceptance at a data level	Interviews that informed web-based questionnaire (low)
Pais et al [54], Switzerland	12-month observational study; to evaluate a new in-home monitoring system among home-dwelling older adults, their family caregivers, and nurses for the support of home care	N=46; 13 older adults, 13 family caregivers, and 20 nurses (characteristics of nurses not given)	In-home monitoring system DomoCare using ambient sensors to monitor mobility, sleep habits, refrigerator visits, and door opening and closing; wearable sensors: activity tracking and ECG^z^	Opinion on the usefulness of ambient and wearable sensors; satisfaction of the older adults, family caregivers, and nurses with ambient and wearable sensors; impact of sensors on the relationships among the older adults, family caregivers, and nurses; impact on in-home care practice (integration and barriers)	Observational study (low)
Peek et al [55], the Netherlands	Qualitative, longitudinal field study; to gain insights into the positions of stakeholder groups involved in the implementation of technology for aging in place	N=29; 5 groups of stakeholders in the process of implementing technology for aging in place: 6 older adults, 7 care professionals, 5 home care and social work managers, 6 technology designers and suppliers, and 5 policy makers; mean age 32.55 years	Scenarios that describe aging in place and the need for creative solutions to provide good-quality care	Types of technologies that could support aging in place; feedback on when participants would consider that the use of technology for aging in place is a success; what participants need to be able to successfully implement technology for aging in place; what participants can contribute to achieve successful implementations	Focus groups (low)
Rampioni et al [56], Italy and Romania	Qualitative study; to collect and analyze the perspectives of older adults, family caregivers, and stakeholders in the fields of care and technology on a list of devices that promote healthy aging	N=30; 13 older adults; 8 caregivers; 9 clinical stakeholders, including 1 end-user representative; and 8 psychologists; 6 women and 3 men	A 15-minute video about the European project SAVE^aa^ was presented; a storyboard was used to show participants how they would interact with the SAVE system; the SAVE system is a multicomponent platform with multiple smart home and wearable sensors streamed directly to a cloud-based platform to detect behavioral and psychological deviation; location services; telemedicine; thermostat; video communications; antiflooding; smart plug (appliances); gas detector; to-do list	Whether the video or storyboards evoked something in their mind and what (features, services, or suggestions to improve the system); why they found it interesting or otherwise, including added value and pains and gains	1:1 interviews with older adults and caregivers; focus group with clinical stakeholders (low)
Verloo et al [57], Switzerland and France	Qualitative; to examine and understand the perceptions of professional caregivers, informal caregivers, and older adults with cognitive impairment by showing photos of different technologies	n=68 CDOAs^ab^ (74% women with a mean age of 82, SD 7.2 years; 60 urban, 35% independent, 34% physically impaired, and 31% cognitively impaired); n=21 IC^ac^ (mean age 68, SD 13.8 years, with 76% being women and retired [n=13]); n=32 PCs (mean age 46.7, SD 9.4 years, mostly female, 91%; physicians, 10%; nurses, 34%; social workers, 6%; nursing assistants, 10%; care assistants, 31%; and OTs, 3%)	10 photographs of relevant smart technologies, including light path, fall detector, electronic pill box, robot vacuum cleaner, service robot, GPS bracelet, touch-screen tablet, social network, brain training, and activity sensor	Perceptions of these technologies among CDOAs with physical and cognitive impairment, professional caregivers, and informal caregivers	1:1 interviews, focus groups, and photo elicitation interviews (low)
Warner et al [58], Canada	Qualitative; apply an implementation science framework to explore the multilevel barriers and facilitators that could affect the implementation of passive remote monitoring technology in home care settings from the perspectives of key informant stakeholders from Nova Scotia, Canada	N=20; 4 policy makers, 4 home care managers, 6 direct care providers, 3 resource navigators (registered nurses), and 3 technology providers	Passive remote monitoring technology, including motion sensors, cameras, and medication administration monitoring	Key informant stakeholders’ perceptions of the barriers to and facilitators of implementing passive remote monitoring technology among older home care service recipients	Interviews (low)

^a^GRADE: Grading of Recommendations, Assessment, Development, and Evaluation.

^b^OT: occupational therapist.

^c^HCP: health care professional.

^d^PT: physical therapist.

^e^CHN: community health nurses.

^f^IWSS: intelligent wireless sensor system.

^g^RCT: randomized controlled trial.

^h^QoL: quality of life.

^i^RN: registered nurse.

^j^WSN: wireless sensor.

^k^LTC: long-term care.

^l^NGO: nongovernmental organization.

^m^HCW: health care worker.

^n^PLWD: persons living with dementia.

^o^AI: artificial intelligence.

^p^Not applicable.

^q^RFID: radio frequency identification.

^r^GP: general practitioner.

^s^IAT: intelligent assistive technology.

^t^IOT: Internet of Things.

^u^PIR: passive infrared.

^v^ODL: observation of daily living.

^w^AT EASE: Automated Technology for Elder Assessment, Safety, and Environment.

^x^PC: professional caregivers.

^y^AAL: ambient assistive living.

^a^ECG: electrocardiogram.

^aa^SAVE: Safety of Elderly People and Vicinity Ensuring.

^ab^CDOA: community-dwelling older adults.

^ac^IC: informal caregivers.

**Table 2:** Summary of key factors that may affect clinician readiness.

Theme and subthemes		Exemplary quotes
**Perceived benefits**		
	Improved clinician-patient relationships	“...Having the ODL data visually available for both the patient and physician can help remove the confrontational aspect of asking the patient about bad habits. Instead, with a shared objective view, the physician can have a conversation about ODL data similar to how she discusses laboratory results with patients.” (Physician; Lee and Dey [49]) “We wouldn’t rely completely [on physical motion information], I’d interview, ask, and listen as well of course.” (Nurse; Klemets et al [48]) “We were hoping it would help them with the assessment process and help them to genuinely understand how people use their homes and therefore what their needs were, so if that person wasn’t really showing, for example, that they were making or seeming to be making themselves regular drinks, that’s something that we would be able to factor in, because sometimes when you speak to someone and they say ‘oh yes I eat very regularly and oh yes I’ve had no trouble at all making a cup of tea,’ but they’re either telling you what they think you want to hear or forgetting or fibbing or something, so we felt it would be a useful kind of tool to help a professional really understand the holistic needs of someone in not too intrusive a way.” (Case study site 2: Participant 04 [41])
	Detection and prediction of health events	“If they found that a client’s feet were swollen, this could be explained by sleeping while sitting in a chair rather than sleeping lying down.” (Nurse; Klemets et al [47]) “...for those that we visit less regularly it would be beneficial, those that are still in good shape. If there is a sudden change, we could find out earlier. Parkinson’s and Alzheimer’s disease have these degradation phases.” (Nurse; Klemets et al [48]) “Reporting on tenant motion within the apartment was therefore deemed to be a useful activity report. Other activity reports requested were, sleep pattern, water usage, front door activity and general activity within the apartment. Staff opinion was that these could be useful on a daily basis to inform the care requirements of individual tenants.” (Dementia care staff member; Martin et al [51]) “...the record provided a ‘suggestion of a problem’ that he did not detect from his visit in the office and made him suspicious about patient’s ‘fishy’ situation. If he had seen these data during the patient’s last office visit, he would have tried to investigate.” (Lee and Dey [49]) “I think it could help patients because it would make them more independent with their care, make them more responsible, and seeing their signs and symptoms will give them the power to manage [them]...and hopefully prevent hospital admissions.” (Participant 4; nurse [46]) “The patient might be thinking, okay, this shortness of breath is [...] probably usual for them, normal for them, but they are not thinking...this shortness of breath may be worse [than usual]. But if it can be picked up with the smart home system, it can be picked up early stage, which can prevent worsening of HF. Preventative measures can be taken before they become really worse when that patient needs the hospitalization.” (Participant 8; cardiologist [46]) “[Remote monitoring technologies]...allows people to respond faster when something goes wrong.” (Key informant 11; home care manager [58]) “Part of the selling point of IndependencePlus was that, you know, the machine learning would pick up when somebody’s daily routine had changed and would alert you to that fact. So, you know, the kit would send a text message to a carer saying ‘Usually your mum has five cups of tea by this point and today she’s only had one,’ you know. ‘Do you want to check this out?’ or, you know, ‘Your mum’s usually out of bed by this time; she hasn’t got up yet, might be worth going round.’” (Case study site 5: Participant 05 [41])
	Facilitation of evidence-based practice	“I think the environment and the type of dementia care...an individualized care closely dependent on the stage of the disease and as adapted as possible to the personal needs (of the user).” (Psychiatrist; Ienca et al [45]) “I would say it’s patient report, when we’re recommending a lot of these things it’s about independence, safety, and reduced caregiver burden, so those are kind of the things I’m looking for, and also are they still using it in 6 months when they come back for their re-eval or did they abandon it because it’s just too complex.” (Practitioner [35]) “I think patients are not aware [...] as to what they need to know and why so it can be hard to drive compliance. Sometimes you have to educate patients multiple times. You know, one individual session just before the patient is being discharged from the hospital is not enough. You have to repeatedly provide the same information to the patient [and] encourage them to ask questions [...] If something like this can be organised, like even if it’s telemonitoring [...] or web monitoring, that would be great.” (Participant 1; nurse [46]) “Within advanced HF, people end up having certain devices implanted, like, cardiac resynchronisation therapy pacemakers. These have different sensors as well as implanted in the heart that give us an idea sometimes of whether the patient is holding on to more fluid and helps doctors adjust things before the patient becomes too symptomatic.” (Participant 2; nurse [46]) “...that client...was supported in his home much longer than anticipated...Because the reason why he would get readmitted [to hospital], have frequent admissions to the hospital, was because he was forgetting to take his medication.” (Key informant 4; home care manager [58])
	Positive impact on patients, clients, and family caregivers	“If those devices weren’t there, I feel that there were some [home care] clients that...would have been normally removed from their home because of the risk...” (Key informant 4; home care manager [58]) “...that there’s...a few long-term care beds that are open, and it’s so difficult to get in. And then seniors often land in the hospital and take up the hospital beds...and I know that there’s a great deal of stress in terms of the number of...workers that are available...the homecare business or support has been very much challenged...homecare may be only able to provide you with two hours a day when really that senior requires more than those two hours.” (Key informant 20; registered nurse [58])
	Peace of mind	“Something like the smart home system, it’s a very [...] efficient module or system, because [...] it helps relax the people at home, the patients themselves and the health personnel. Because they know that they are taking care of the patients, [...] even though if he’s very far away, they know that he’s safe because you can see all the data.” (Participant 3; nurse [46]) “This allows them...to stay at home with that peace of mind, especially their families to know if they’ve had a fall. That’s a big reason why people end up going to long term care sooner, is if they’ve had frequent falls, if they’re not safe at home.” (Key informant 13; direct care provider [58])
**Perceived barriers**		
	Impact on clinicians	“A presently trained and configured family...doctor would think it was junk. They would go, ‘Well, that’s interesting, I got 9 more minutes...[of the appointment left].’” (Physician; Beaudin et al [32]) “Nurses claimed that they were too busy in their daily work and that the many other computer systems they already had to use were too time-consuming.” (Nurses; Klemets et al [47]) “Db6 an oncology specialist, expressed that he (and his office staff) would be too busy to review charts of the ODL data before a visit with the patient because they are already overloaded with tasks to perform.” (Physician; Lee and Dey [49]) “People in the clinics have just a general idea of what can be done, but very few ideas, not so much understanding of what that technologically means.” (Psychiatrist; Lenca et al [45]) “I don’t have much knowledge about this. It’s true and it makes sense that some training on this wouldn’t be bad.” (Professional caregiver; Verloo et al [57]) “It’s a lot of trial and errors so often times I have to go back to the assessment part of things after trying 1 device that doesn’t work, so it’s kind of ongoing throughout the course of treatment.” (Practitioner 45) “Yeah, there still isn’t a road map. There are no instructions on how to do this. There’s no textbook. And oftentimes while manufacturers may make their products compatible, they don’t tell you how to combine them...” (Practitioner 45) “Our lead domiciliary care provider, they’re not geared up to looking at health data and making health judgements based on that, so quite rightly they were saying ‘we’ve got this thing that says heart rate spike, what does that mean, do we have to contact a GP, what’s going on?’ So there was a lot of confusion around that, and we actually stopped using the system through COVID because of that.” (Case study site 4: Participant 01 [41]) “Because so much of it is health metrics or what would indicate medical problems or something that needs medical attention and support, I think that it needs to be the team that knows the most about that information, it needs to be people who can interpret what normal heart rate data needs to look like and what normal sleep patterns might look like...so it needs people who know what they’re looking at, know how to interpret it and are skilled and already knowledgeable about how to take that medical data and turn it into actions. This is when we need to call the GP. This is when we need to call an ambulance. This is when we need to change these meds. This is when we need to—you know, it needs to be the people who will make those medical decisions who are interpreting those data.” (Case study site 1: Participant 01 [41])
	Impact on clinician identity	“...worried that if he had been given the ODL data, a jury one day may question his ‘interpretation of the dots’ in the visualization if the patient had an adverse event related (or even worse, unrelated) to the data in question.” (Physician; Lee and Dey [49]) “I think that these instruments should remain assistive tools and shouldn’t replace medical examinations, diagnoses or therapies. I find this a risky trend: if doctor-patient contact is abolished and everything runs via apps...I think this is dangerous...” (Psychiatrist; Lenca et al [45]) “Some night staff felt that the RFID [location-based tracker] system had been used as a ‘Big Brother tool for management’ to monitor staff activity.” (Health care worker 48) “I think it’s hard to keep up with all the technology changes and I find that as soon as I learn something new, my patients have surpassed me or their family members have heard of something...” (Practitioner 45) “The problem is people probably want all of it and they can’t have all of it, so you need to decide what’s your highest priority. Is our highest priority to know when somebody’s fallen over so we can go and pick them up and maybe get them to hospital, is that our highest priority? I don’t know...Or is our highest priority to have lots of data about people so when we come to review them or assess them, we make better decisions?...Or is our priority something as simple and practical as I want a very good automated meds dispenser for people who are able to take their own medication because overnight that saves me about 500 hours of care a week and, what’s that, a lot of money?” (Case study site 4: Participant 02 [41]) “We’re changing the way [we provide] care and that needs some different mindsets and skills or additional to what the care staff have. So the care staff generally have very—they want to be supportive and help people—they’re that kind of character generally—some more than others are comfortable around technology.” (Case study site 1: Participant 02 [41])
	Potential adverse impact on the patient	“These are people that no longer use any technology in their daily life, except for a light switch...very few can use a coffee machine, so it’s very difficult to approach...” (Gerontologist; Ienca et al [45]) “What worries me is situations like this, where the children have even put cameras in the bedroom, not to monitor their parents, but rather to reassure themselves.” (Professional caregiver; Verloo et al [57]) “...there is something about, as I am saying, when I enter a patient room then there is something about what I see and smell and find out how things are as a whole, plus he [the patient] might say that today I would like to watch TV a bit longer...for example.” (Health care provider; Nilsen et al [39]) “...it should be person-centred, and technology isn’t person-centred...you’ve got the technology, but you can’t use it until he’s capable of accepting [it]...you can’t treat everybody the same, and that’s where technology falls down, because it’d be too [expensive] to personalise it, and then who’d pay for that?” (Nurse 48) “...these technologies could like bring on a sense of paranoia or bring on some behavioural and psychological symptoms of dementia for someone...oftentimes people talk about being watched, and we brush that off as being a sign of dementia. When in this case...It would be accurate.” (Key informant 12; direct care provider [58]) “I often explain it that we used to, when we were going out for like IndependencePlus and other technology projects in the past, we used to think what technology’s out there and let’s go and buy it and now let’s look at people that we provide care for and fit them to that technology. That is probably the biggest learning, that that’s a mistake. We shouldn’t be doing that.” (Case study site 4: Participant 01 [41])
	Concerns about privacy and data security	“...there are ethical problems if it’s used as a means to monitor the person...” (Professional caregiver; Verloo et al [57]) “I have no problem displaying what I do at work. I rather think of the user, of...Where did the privacy go? I enter and leave the room and do my job, and am supposed to be professional. But the users shall feel that they have a private life when they enter their flat, that they are not going to be under surveillance, ’cause that is unnatural.” (Health care provider; Nilsen et al [39]) “...we get lots of issues or questions from folks around privacy...” (Practitioner 45) “...going back to the whole privacy thing as well, that’s very important to clients. So educating them about how this technology works...” (Practitioner 45) “The privacy and confidentiality issue will be very important for the patient, especially when we’re talking about clouds and everything and how to protect their privacy.” (Participant 6; pharmacist [46]) “The challenge with this, even as a concept, is this idea of it’s all a bit Big Brother-like, it’s all a bit you know, sort of a bit ‘spying’...The challenge would be to break down some of the stigma that might come with that. I’m not necessarily saying that it’s true, that it’s like a bit Big Brother-like, but I think that is the perception amongst some people who might be resistant to using the technology, you know. If it’s just there forever, recording how many times I use the toilet, you know, it’s uncomfortable.” (Case study site 2: Participant 03 [41])

### Outcomes Targeted

Clinician feedback, attitudes, perceptions, and experiences regarding the use of HAS technology is critical because clinicians are an important end-user group [46,58,59]. Clinicians may use the technology to monitor people living with chronic diseases [46] and use the information in care planning [13]. While the smart technology could optimize clinical care delivery and augment clinical decision-making, certain factors may impact clinicians’ readiness to adopt smart home technology for health monitoring*.*

### Populations Included in the Review

Among the 27 studies included, most (n=19, 79%) had a mix of clinical and nonclinical participants, including social workers [32-34,43,44], management [33,35,45,50,51,55,58], policy makers [55,58] or decision makers [41], aging-in-place specialists or experts [35,40,41], older adults [40,42,50,55-57], relatives [42,52], informal caregivers [56,57], and technology designers and suppliers [35,41,55,58]. Table 1 shows that the clinician population recruited was highly variable. Health care workers and care staff and support workers were included in several studies [32-34,37,38,40-42,44,51,53,55,57,58]. The represented clinical disciplines included nursing [33,37,38,40,41,43,44,46-48,58], medicine [32,38,39,43,44,46,49,50,52,53], physiotherapy [34,35,37,43,44], ergotherapy [37], cardiology [46], occupational therapy [35,43,44], psychology [33,56], psychiatry [45], pharmacy [46], gerontology and geriatrics [45], speech and language pathology [35], and neurology and neuropsychology [45,50]. Clinicians worked in a variety of settings in various roles, including in community settings such as home care as direct care providers, navigators, or managers.

### Smart Home Technology Use

The studies described a variety of smart home–type technology to monitor specific aspects of activities of daily living. The use of mainstream smart home technology such as home automation devices and smart speakers was described [35], and other studies (n=14, 52%) reported on the use of technologies for remotely monitoring several aspects of activities of daily living, including appliance use [41,55]; use of water [51]; medication adherence [47,49,55,57]; phone and coffee maker and kettle use [41,49]; bathroom and toilet visits [41]; time spent in different rooms and time spent outside [48]; cooking [51]; nighttime monitoring of resident location [38,39,42,52]; general in-home physical motion [57]; resident mobility, including steps ambulated, such as location, activity time, and duration [42]; door use and opening [39,41,42,51,52]; health issues [36,55]; fall detection [34,55,57]; wandering detection [55]; and monitoring of getting in and out of bed [39,42,46]. Several studies (n=6, 22%) included the use of cameras or microphones [33,53], wearables to track activity [46,54], and electrocardiograms to monitor heart rate connected to web-based portals and mobile devices [39]. Pressure mats [42], smart beds [40], security blankets [39], and alarms [52] were used to monitor residents’ activities of daily living, such as sleep and other activities, and remind them to engage in health activities (eg, taking their medication and sleep hygiene). Telecare and remote coaching to facilitate communication were also reported [33,39,52]. Authors described monitoring of task-based resident in-home activities [49] to facilitate residents’ independence and emotional connectivity while minimizing staff intrusions [37,51]. For example, in the study by Glasby et al [41], the authors report the implementation of home-based sensors with artificial intelligence capabilities in pilot case study sites. The sensors were placed in key locations of individual homes to remotely monitor opening and closing of doors, kettle use, refrigerator door opening and flushing of the toilet [41].

Notably, 30% (8/27) of the studies did not implement the smart home technologies but investigated health care professionals’ predeployment preferences for using smart home technologies [34,43,44,46,56,58], privacy concerns [33], and the issues associated with using smart home and telemonitoring systems [43,44,46]. For example, to obtain clinicians’ perspectives, Islam et al [46] provided the clinicians involved in the clinical care of patients with cardiovascular disease with a schematic for a prototype smart home system that included sensor-enabled medication monitoring; blood pressure, weight, and fat percentage monitoring; and a wearable for sleep and heart rate, as well as educational content, alerts, and messaging [46].

In another study, Warner et al [58] explored key stakeholders’ perceptions of the barriers to and facilitators of implementing passive remote monitoring technology, including motion sensors, cameras, and medication administration sensors among older home care service recipients [58].

Similarly, the studies by Hunter et al [43,44] investigated the issues that are potentially associated with using smart home and telemonitoring technologies to support older adults. The work by Hunter et al [43,44] was first reported as a research brief [43] and later as a traditional research paper [44]. Another study explored whether sensor-generated motion patterns could support the work of nurses [48] and elicited clinician’s views on the sensor-generated data visualization displays. The study by Beaudin et al [32] examined the types of physiological indicators that clinicians would like to monitor for the patients in their care [32]. The study by Rampioni et al [56] examined stakeholder perspectives on how information and communications technologies and sensing technologies could address the needs of older adults for active and healthy aging [56].

## Discussion

### Principal Findings

#### Perceived Benefits

Many clinicians in the studies included in this systematic review recognized the benefits of integrating smart home technology to monitor and facilitate care of their patients. First, clinicians perceived that overall quality of care can be enhanced with the use of a variety of patient data collected objectively and over time by the sensor technology [32,34,43,45,47-49,54]. For example, previously difficult-to-obtain data such as nighttime behaviors can provide accurate information about a patient’s well-being that may otherwise not be included in clinical decision-making [47]. In addition, there is often a mismatch between what patients report and what is actually happening in the home. Understanding the actual holistic needs of patients as unobtrusively as possible was considered helpful for health professionals [41]. Second, clinicians perceived that adopting smart home technology for remote health monitoring could lead to feeling more informed by real-time and just-in-time data [34,46-49]. Provision of just-in-time data can facilitate timely patient transitions [34], allow for appropriate clinical responses [34,47,48], and enable clinicians to act quickly in response to data that could forecast clinical emergencies [46,53,58]. Third, clinicians recognized that the combination of access to real-time, objectively collected data can facilitate clinicians’ understanding of patients’ needs and risks and tailoring of care approaches accordingly [46,53,58].

Fourth, clinicians perceived that access to data about activities of daily living collected in the context of the patients’ environment was beneficial because it could empower clinicians to identify early patterns of decline [32,34,47,48] and changes in cognitive and functional capacity [34,47-49], forecast future health events [46,47], and enable clinicians to tailor appropriate actions to address health concerns [32,49]. In addition, in-context sensor data [32] were considered more reflective of a patient’s health, which could empower clinicians to implement and evaluate tailored care approaches and identify and act upon any issues quickly [32,34,48]. Importantly, contextually collected smart home sensor data case studies could be useful to promote adoption of a specific intervention or care approach [49,58].

A final benefit perceived by clinicians was the potential for enhancing clinician-patient relationships and the ensuing positive psychosocial impact on patients. Clinicians recognized the potential of smart home technology to streamline clinical visits, saving clinicians and patients time [43,58] and reducing health care costs [45,50]. Importantly, clinicians could present the collected data to patients in the form of a visual summary [49], which could facilitate meaningful discussions with patients or family members about their care [32,51,58], enabling patient-centered, informed care decisions [48]. Smart home technology–enabled patient-centered care could support clinicians in addressing numerous dimensions of care concerns, such as respect for the values, preferences, and needs of patients; coordination of care and integration of services; communication and accurate, timely, and appropriate information; enhanced physical comfort and emotional support; involvement of family and friends; and transition and continuity of care [58,60]. Addressing these dimensions of care can lead to strong clinician-patient relationships, which can empower patients to feel more involved in their care [61,62] and self-management [46,56]. Consequently, contrary to perceived threats of smart home technology resulting in disconnection between clinicians and patients [39,57], many clinicians reported that the use of smart home technology for remote health monitoring could strengthen clinician-patient relationships and enhance patient motivation [32,50] and social relations [45].

#### Perceived Challenges

Clinicians perceived several factors that were challenges to the adoption of smart home technology for remote health monitoring. Chief concerns included privacy, data security, and the ethical and moral use of potentially “invasive” technology and the clinicians’ capacity to learn how to use the smart home technology and the generated data. First, nearly half (11/27, 41%) of the reviewed studies highlighted clinician concerns about the privacy and dignity of patients and the security of their data [32-35,39,42-44,46,47,51,53,57]. Brand et al [33] provided insight into the privacy concerns of a mix of stakeholders, including nonclinicians. Survey respondents emphasized the importance of both patient privacy and the secure storage of personal data. They were particularly concerned with the use of video cameras with visual fields that included bedroom and bathroom entrances [33]. While such locations within the home are essential for establishing and monitoring important patient norms regarding activities of daily living [63] and well-being [64], in contrast, Nilsen et al [39] acknowledged that monitoring using a digital night surveillance system with digital communication, sensors on doors, and electronic security blankets made it possible to monitor the patient without disturbing them [39]. These findings suggest that clinicians may perceive sensor technology as less intrusive than video cameras. In addition, clinicians who experienced using the smart home technology highlighted that physically entering a patient’s room was also an invasion of privacy [39]. Concerns were also raised regarding the privacy and ethical use of sensor-collected data by Peek et al [55] and Islam et al [46]. These authors emphasized the importance of confidentiality and protecting patients’ privacy via clear privacy policies when such technology is used. This suggests that both clear policies and a working knowledge of the available smart home sensor technology will be important for its successful adoption by clinicians.

Second, clinicians expressed concerns regarding their capacity to successfully adopt smart home technology, highlighting the demands required to master software and build the requisite competency [35,43,54] and the perceived time required to view and interpret data [47,54]. A lack of familiarity with sensor technology was a clear challenge [39], highlighted by clinicians citing the effort required to “get used to the system deployed” and “understand and interpret graphs” [47]. Notably, Ienca et al [45] reported a lack of clinician technical skills and digital resources as perceived causes of suboptimal care. While clinicians perceived the need to gain competency regarding smart home technology and the interpretation of smart home sensor data as a challenge, clinicians have been described as capable of understanding the depth of information derived from smart home technology over time [47].

In addition, while older adults enjoyed using smart home technology [55], clinicians expressed concerns about patient readiness to adopt complex smart home technology [44]. Similarly, Ding et al [35] explored the service delivery practices of mainstream smart home technology as assistive technology and found that there were concerns about lack of instructions or manufacturer guidelines on the compatibility and interoperability of devices.

Subsequently, the use of mainstream smart technology in combination with other health-related smart home technology may encounter barriers to adoption by clinicians. Clinicians may need to anticipate substantial education and knowledge gaps, especially among patients who may have concerns about Big Brother–type intrusion into their private life [43,58]. The potential deficits (skills and resources) of both clinicians and patients will need to be addressed for broad adoption. Deployment of a user-friendly and easily navigated system [51,55] could facilitate successful adoption and ensure optimal provision of care.

The perceived negative impact of smart home technology on patient well-being is another important factor that could impact clinician readiness to adopt from psychosocial, quality of care, and equity perspectives. Clinicians reported perceptions that smart home technology could be harmful to patients [58,65]. For example, patients may incorrectly interpret their sensor data assuming causation rather than correlation between behaviors and changes in health [32]. This could lead to self-directed lifestyle changes to the potential detriment of their overall health and quality of life [32,39]. In another example, Warner et al [58] found that patients may experience paranoia because of monitoring devices in the home.

Similarly, clinicians recognized that potential evidence of a decline in health could lead to depression or have a negative self-fulfilling effect on a patient’s self-perception [32], exacerbating their physical and mental conditions. Clinicians also raised concerns that remote monitoring may create real or imagined distance between themselves and their patients, which could have a negative impact on the health of the patient [39,57]. For example, many patients placed a high value on nurse-patient interactions [60]. Conversely, the question of equity of access was also raised by some clinicians, posing a social justice challenge to adoption wherein some patients may benefit from access to the technology while it remains inaccessible to others [39], thereby making it inequitable for clinicians to adopt smart home technology for certain segments of the population (eg, those who can afford it). All these factors regarding the well-being of patients expressed by clinicians need to be considered because they could impact clinicians’ readiness to adopt smart home technology [55].

Finally, some clinicians in the reviewed studies perceived the use of smart home technology as a threat to their professional culture, highlighting both logistic concerns and misconceptions about this technology [39,42,45,57]. In contrast to the perceived benefits of deploying smart home technology, some clinicians raised concerns that it may replace the role of some allied health professionals and create distance with patients [34]. Others expressed concern about the clash of professional cultures [39], emphasizing that technology cannot replace human-delivered person-centered care [39,42,45,57]. Some participants voiced concerns about potential incongruity between health care and IT and business priorities [39,42,45]. Such views highlight an important misconception and knowledge gap regarding smart home technology—that it is deployed to *replace* clinician contact with patients. However, the opposite is true if smart home technology can *enhance* clinician-patient communication [32,51] and enable clinicians to better understand their patients’ needs [49] and quickly respond [32,34,47]. Previous negative experiences with technology may exacerbate such misconceptions, highlighting the need for clinician-driven technology showcases and peer-reviewed evidence to establish the credibility, system reliability, and clinical validity of smart home technology [45].

A well-designed co-creation process involving key professional stakeholders can help address negative experiences, the incongruity between health care and IT priorities, and reported logistical concerns regarding time and money cost [35,39,58]. Theoretical models to help advance clinician readiness for smart home adoption could provide strategies that address or mitigate challenges that negatively impact the readiness for adoption. The findings of this systematic review suggest that there are several factors that impact clinician readiness to adopt smart home technology. The factors related to using smart technology and interventions for remotely monitoring patient health and well-being were categorized into perceived challenges and benefits.

If widespread adoption of health smart homes is the goal, a model is needed to facilitate the co-design of technology use, support its implementation, and ensure its ethical use. Figure 1 is a proposed theoretical model that could enable clinicians to weigh the benefits and challenges, including potential ethical issues of smart home implementation.

The first step in the model is to provide clinicians with opportunities to acknowledge both the challenges and potential benefits of smart home technology for remote health monitoring. Discussing potential benefits alone is not nor should be sufficient to convince clinicians to adopt smart home technology. For widespread adoption, potential challenges will need to be practically discussed and addressed. For example, the use of remotely collected sensor data on activities of daily living is a new form of evidence that clinicians will need to learn to use for evidence-based practice. Clinicians will need to have confidence that they will receive training on how to optimize their clinical decision-making using their patients’ sensor–collected smart home data [56].

Second, the experiences of early adopters [66] with smart home technology could be useful for clinicians because early adopters can acknowledge challenges, provide education on potential misconceptions, and affirm that clinicians are patient advocates. Early adopters can present the benefits of adopting this technology by showcasing the findings and lessons learned of real-world small- or larger-scale smart home technology implementation. Third, researchers [8] have an important role in facilitating clinician adoption through the co-creation process with clinicians, IT experts, business logistics professionals, patients, and patient advocates that includes the development or refinement of smart home technology, conduct of studies, development of policies, and creation of new smart home technology–enabled care paradigms. The dissemination of small- and large-scale implementation study findings and the presentation of data-driven case studies can collectively provide valuable, pragmatic evidence; critical evaluation; and commentary in peer-reviewed manuscripts. The dissemination of such evidence is useful to enhance clinician readiness and would support eventual widespread adoption of smart home technology for remote health monitoring as a standard of care for older adults who wish to age in place. Finally, reports of experiences of the co-creation process, such as that proposed by Nilsen et al [39] and Islam et al [46] and exemplified by the study by Glasby et al [41], are invaluable as they signpost a practical pathway to adoption and validate the clinical relevance of the purpose of smart home technology to support older adults.

### Implications for Clinicians

More studies are showing that both older adults and family caregivers are open to adopting smart homes to facilitate and support aging in place [34,54,67]. It will be important for the members of the multidisciplinary health team to become knowledgeable of smart home technology and how it can be used to augment traditional care models by remotely monitoring health and well-being and supporting efficient and effective care coordination for the older person [51,59]. Although several different health disciplines were part of the studies in this review, it is clear that there are major differences in clinician expectations of the HAS. For example, home health nurses may be interested in smart home data to ensure that the older person is performing their usual daily routines, whereas a primary care physician may be more interested in certain data points that could help with early identification of a change in health. In addition, very little is known about how the different disciplines may envision the integration of the HAS into their clinical practice and the changes needed in the current systems and processes for the translation of the HAS to support the health and wellness of older adults living at home.

To benefit clinical care, clinicians will need to learn some technical and practical aspects of how to use the data collected by the smart home system that may be deployed in the homes of clients whom they are caring for. Accordingly, it is conceivable that the ever-evolving roles of clinicians will include leading change in the digital health space [50]. It is likely that workloads will be impacted; however, more knowledge is needed to understand how to integrate the smart home system into new “smart” models of care and how to use the sensor data to support clinical decision-making. The cost-benefit of this technology as a health care solution, including impacts on staffing shortages and care delivery in rural and remote locations, also needs to be investigated. Finally, for clinicians to embrace the artificial intelligent agent as a new member of the health care team, and to optimize the smart homes’ capability, clinicians need to be offered training specifically focused on the use of smart home technology and sensor data interpretation. Tertiary education frameworks need to include these monitoring technologies as a core competency.

### Limitations

This review demonstrates that the scientific understanding of clinician readiness to adopt smart home technology is progressing. The studies reviewed were varied and all studies included a range of stakeholders with relatively small sample sizes, making it challenging to synthesize the findings for specific clinical disciplines. The diversity in study populations, which often included multiple types of clinicians and other stakeholders, makes it difficult to extract findings that are directly applicable to any single clinical discipline.

Only English-language papers were included; however, during our search, we did not encounter non–English-language papers. While qualitative studies with relatively small sample sizes are often ranked lower in traditional evidence hierarchies compared with RCTs, which are considered the “gold standard,” qualitative research provides essential insights into user experiences that are crucial for designing effective, user-centered technologies and the policies and procedures needed for implementation. Dismissing these contributions in favor of a rigid adherence to RCTs overlooks critical aspects of practical implementation and user acceptance that determine the real-world success of interventions. Moreover, the rapid pace of technological development necessitates timely research to keep up with innovations, suggesting that waiting for large-scale RCTs (or waiting for the funding to execute such studies) might render findings obsolete by the time they are applied or indeed stifle implementation in new models of care. The integration of smart home technology into models of care for older adults has been challenging, requiring researchers to partner with a variety of clinical and nonclinical professionals, such as technologists, designers, clinicians, and business and industry professionals, to study various aspects of co-design, acceptability, and implementation. This could explain why researchers have used a more pragmatic approach for their studies, including nonprobability sampling, purposive and convenience sampling, case studies, and multiple sources of data.

Another major drawback to synthesizing the literature is the incongruence of the definitions used for smart home technology and the fact that most studies included a mix of clinicians and nonclinicians. As clinicians represent a major end-user group, they need to be more involved in smart home co-design and implementation studies. More studies that focus on specific clinician groups (eg, nurses and physicians) are needed to determine the specific factors that could potentially undermine the success of smart home monitoring for patients at home. Finally, the COREQ (Consolidated Criteria for Reporting Qualitative Research) guidelines should be used to report comprehensive qualitative findings. While the conduct of RCTs may not be sufficiently pragmatic for this field, the use of mixed methods could yield more generalizable findings. The studies by Delbreil and Zvobgo [37], Elavsky et al [40], and Offerman-van Heek and Ziefle [53] are all good examples of providing readers with greater depth of knowledge regarding clinicians’ readiness.

### Conclusions

This systematic review synthesized the literature on clinician readiness to adopt HAS technology for remote health monitoring—a key determinant for the future successful adoption of such technologies. The review identified several factors that may impact readiness for adoption among clinicians, highlighting both challenges and perceived benefits of smart home technology. The ensuing proposed theoretical model outlines the steps and key roles that early adopting clinicians, researchers, and cocreating stakeholders will have if widespread clinician adoption of smart home technologies for health monitoring occurs. Importantly, an incongruence in the terminology used in the limited body of literature to date reinforces the need for standardized reporting when discussing smart home technology to develop a clear and consistent body of credible evidence. Future research should continue to evolve this body of literature, evidencing the credibility, reliability, and clinical validity of smart home technology through a combination of peer-reviewed case studies, well-designed research trials, and commentaries. Continuing to reflect, act, and report on clinician’s experiences of smart home technology will help identify and overcome challenges to adoption and simultaneously affirm and embrace its benefits. Doing so can establish the professional acceptability and clinical validity of this technology, underpinning its widespread adoption in clinical practice.

## Appendix

Multimedia Appendix 1 Themes and subthemes.

Multimedia Appendix 2 Prisma checklist.

## Abbreviations

COREQ: Consolidated Criteria for Reporting Qualitative Research
GRADE: Grading of Recommendations, Assessment, Development, and Evaluation
HAS: health-smart home
JBI: Joanna Briggs Institute
PRISMA: Preferred Reporting Items for Systematic Reviews and Meta-Analyses
RCT: randomized controlled trial
TAM: technology acceptance model
