# Supplementary material for: Factors Affecting Clinician Readiness to Adopt Smart Home Technology for Remote Health Monitoring: Systematic Review
Source: JMIR Aging. 2024 Dec 5;7:e64367. doi: 10.2196/64367 (PMC11659702; doi:10.2196/64367)
Supplement: Multimedia Appendix 1 [file aging_v7i1e64367_app1.docx]

Multimedia Appendix 1: Descriptive Themes and Sub-Themes

| Themes | Subthemes | Exemplar Quotes |
| --- | --- | --- |
| Perceived Benefits | Improved clinician-patient relations | *‘…Having the ODL data visually available for both the patient and physician can help remove the confrontational aspect of asking the patient about bad habits. Instead, with a shared objective view, the physician can have a conversation about ODL data similar to how she discusses laboratory results with patients.’*  [Physician, Lee 2015]  *‘We wouldn’t rely completely [on physical motion information], I’d interview, ask, and listen as well of course.’*  [Nurse, Klements 2017]  *‘We were hoping it would help them with the assessment process and help them to genuinely understand how people use their homes and therefore what their needs were, so if that person wasn’t really showing, for example, that they were making or seeming to be making themselves regular drinks, that’s something that we would be able to factor in, because sometimes when you speak to someone and they say ‘oh yes I eat very regularly and oh yes I’ve had no trouble at all making a cup of tea’, but they’re either telling you what they think you want to hear or forgetting or fibbing or something, so we felt it would be a useful kind of tool to help a professional really understand the holistic needs of someone in not too intrusive a way.’*  [CS2 P04, Glasby, 2023] |
|  | Detect and Predict health events | *‘If they found that a client's feet were swollen, this could be explained by sleeping while sitting in a chair rather than sleeping lying down.’*  [Nurse, Klements 2019]  *‘for those that we visit less regularly it would be beneficial, those that are still in good shape. If there is a sudden change, we could find out earlier. Parkinson’s and Alzheimer’s disease have these degradation phases.’*  [Nurse, Klements 2017]  *‘Reporting on tenant motion within the apartment was therefore deemed to be a useful activity report. Other activity reports requested were, sleep pattern, water usage, front door activity and general activity within the apartment. Staff opinion was that these could be useful on a daily basis to inform the care requirements of individual tenants.’*  [Dementia Care Staff, Martin, 2007]  *‘…the record provided a “suggestion of a problem” that he did not detect from his visit in the office and made him suspicious about patient’s “fishy” situation. If he had seen these data during the patient’s last office visit, he would have tried to investigate.’*  [Lee 2015]  *‘I think it could help patients because it would make them more independent with their care, make them more responsible, and seeing their signs and symptoms will give them the power to manage [them]… and hopefully prevent hospital admissions.’*  *[Participant 4, Nurse, Islam, 2022]*  *‘The patient might be thinking, okay, this shortness of breath is (…) probably usual for them, normal for them, but they are not thinking… this shortness of breath may be worse [than usual]. But if it can be picked up with the smart home system, it can be picked up early stage, which can prevent worsening of HF. Preventative measures can be taken before they become really worse when that patient needs the hospitalization.’*  *[Participant 8, Cardiologist, Islam, 2022]*  *[Remote monitoring technologies]’…allows people to respond faster when*  *something goes wrong.’*  *[KI 11, Home care manager, Warner, 2023]*  *‘Part of the selling point of IndependencePlus was that, you know, the machine learning would pick up when somebody’s daily routine had changed and would alert you to that fact. So, you know, the kit would send a text message to a carer saying ‘Usually your mum has five cups of tea by this point and today she’s only had one’, you know. ‘Do you want to check this out?’ or, you know, ‘Your mum’s usually out of bed by this time; she hasn’t got up yet, might be worth going round.’*  *[CS2 P05, Glasby, 2023]* |
|  | Facilitate evidence-based practice | *‘I think the environment and the type of dementia care… an*  *individualized care closely dependent on the*  *stage of the disease and as adapted as possible to*  *the personal needs (of the user).’*  [Psychiatrist, Ienca 2018]  *‘I would say it’s patient report, when we’re recommending a lot of these things it’s about independence, safety, and reduced caregiver burden, so those are kind of the things I’m looking for, and also are they still using it in 6 months when they come back for their re-eval or did they abandon it because it’s just too complex.’*  [Practitioner, Ding, 2023]  *‘I think patients are not aware (…) as to what they need to know and why so it can be hard to drive compliance. Sometimes you have to educate patients multiple times. “You know, one individual session just before the patient is being discharged from the hospital is not enough. You have to repeatedly provide the same information to the*  *patient [and] encourage them to ask questions (…) If something like this can be organised, like even if it’s telemonitoring (…) or web monitoring, that would be great.’*  [Participant 1, Nurse, Islam, 2022]  *‘Within advanced HF, people end up having certain devices implanted, like, cardiac resynchronisation therapy pacemakers. These have different sensors as well as implanted in the heart that give us an idea sometimes of whether the patient is holding on to more fluid and helps doctors adjust things before the patient becomes too symptomatic.’*  [Participant 2, Nurse, Islam, 2022]  *‘…that client…. was supported in his home much longer than anticipated... Because the reason why he would get readmitted [to hospital], have frequent admissions to the hospital, was because he was forgetting to take his medication.’*  [KI 4, Home care manager, Warner, 2023] |
|  | Positive impact on patients/clients/family caregivers | *‘If those devices weren’t there, I feel that there were some [home care] clients that…would have been normally removed from their home because of the risk…’*  *[KI 4 – Home care manager, Warner, 2023]*  *‘…that there’s…a few long-term care beds that are open, and it’s so difficult to get in. And then seniors often land in the hospital and take up the hospital beds…and I know that there’s a great deal of stress in terms of the number of… workers that are available…the homecare business or support has been very much challenged… homecare may be only able to provide you with two hours a day when really that senior requires more than those two hours.’*  *[KI 20, Registered Nurse, Warner, 2023]* |
|  | Peace of mind | *‘Something like the smart home system, it’s a very (…) efficient module or system, because (…) it helps relax the people at home, the*  *patients themselves and the health personnel. Because they know*  *that they are taking care of the patients, (…) even though if he’s very*  *far away, they know that he’s safe because you can see all the data.’*  [Participant 3, Nurse, Islam, 2022]  *‘This allows them… to stay at home with that peace of mind, especially their families to know if they’ve had a fall. That’s a big reason why people end up going to long term care sooner, is if they’ve had frequent falls, if they’re not safe at home.’*  [KI 13, Direct care provider, Warner, 2023] |
| Perceived Barriers | Impact on clinician | *‘A presently trained and configured family…doctor would think it was junk. They would go, “Well, that's interesting, I got 9 more minutes…* [of the appointment left]’  [Physician, Beaudin 2006]  *‘Nurses claimed that they were too busy in their daily work and that the many other computer systems they already had to use were too time-consuming.'*  [Nurses, Klements 2019]  *‘Db6 an oncology specialist, expressed that he (and his office staff) would be too busy to review charts of the ODL data before a visit with the patient because they are already overloaded with tasks to perform.’*  [Physician, Lee 2015]  *‘People in the clinics have just a general idea of what can be done, but very few ideas, not so much understanding of what that technologically means.’*  [Psychiatrist, Ienca 2018]  *‘I don’t have much knowledge about this. It’s true and it makes sense that some training on this wouldn’t be bad.’*  [Professional caregiver, Verloo 2020]  *‘It’s a lot of trial and errors so often times I have to go back to the assessment part of things after trying 1 device that doesn’t work, so it’s kind of ongoing throughout the course of treatment.’*  *[Practitioner, Ding, 2023]*  *‘Yeah, there still isn’t a road map. There are no instructions on how to do this. There’s no textbook. And oftentimes while manufacturers may make their products compatible, they don’t tell you how to combine them…’*  *[Practitioner, Ding, 2023]*  *‘Our lead domiciliary care provider, they’re not geared up to looking at health data and making health judgements based on that, so quite rightly they were saying ‘we’ve got this thing that says heart rate spike, what does that mean, do we have to contact a GP, what’s going on?’ So there was a lot of confusion around that, and we actually stopped using the system through COVID because of that.’*  *[CS4 P01, Gasby, 2023]*  *‘Because so much of it is health metrics or what would indicate medical problems or something that needs medical attention and support, I think that it needs to be the team that knows the most about that information, it needs to be people who can interpret what normal heart rate data needs to look like and what normal sleep patterns might look like… so it needs people who know what they’re looking at, know how to interpret it and are skilled and already knowledgeable about how to take that medical data and*  *turn it into actions. This is when we need to call the GP. This is when we need to call an ambulance.* *This is when we need to change these meds. This is when we need to – you know, it needs to be the people who will make those medical decisions who are interpreting those data.’*  *[CS1 P01, Gasby, 2023]* |
|  | Impact on clinician identity | *‘...worried that if he had been given the ODL data, a jury one day may question his “interpretation of the dots” in the visualization if the patient had an adverse event related (or even worse, unrelated) to the data in question.’*  [Physician, Lee 2015]  *‘I think that these instruments should remain assistive tools and shouldn’t replace medical examinations, diagnoses or therapies. I find this a risky trend: if doctor-patient contact is abolished and everything runs via apps… I think this is dangerous…’*  [Psychiatrist, Ienca 2018]  *‘Some night staff felt that the RFID [location-based tracker] system had been used as a “Big Brother tool for management” to monitor staff activity.’*  [Healthcare worker, Hall 2019]  *‘I think it’s hard to keep up with all the technology changes and I find that as soon as I learn something new, my patients have surpassed me or their family members have heard of something …’*  [Practitioner, Ding, 2023]  *The problem is people probably want all of it and they can’t have all of it, so you need to decide what’s your highest priority. Is our highest priority to know when somebody’s fallen over so we can go and pick them up and maybe get them to hospital, is that our highest priority? I don’t know… Or is our highest priority to have lots of data about people so when we come to review them or assess them, we make better decisions?... Or is our priority something as simple and practical as I want a very good automated meds*  *dispenser for people who are able to take their own medication because overnight that saves me about 500 hours of care a week and, what’s that, a lot of money?*’  [CS4 P02, Glasby, 2023]  *‘We’re changing the way [we provide] care and that needs some different mindsets and skills or additional to what the care staff have. So the care staff generally have very – they want to be supportive and help people – they’re that kind of character generally – some more than others are comfortable around technology.’*  [CS1 P02, Glasby, 2023] |
|  | Potential adverse impact on patient | *‘These are people that no longer use any technology in their daily life, except for a light switch…very few can use a coffee machine, so it’s very difficult to approach...’*  [Gerontologist, Ienca 2018]  *‘What worries me is situations like this, where the children have even put cameras in the bedroom, not to monitor their parents, but rather to reassure*  *Themselves.’*  [Professional caregiver, Verloo 2020]  *‘there is something about, as I am saying, when I enter a patient room then there is something about what I see and smell and find out how things are as a*  *whole, plus he [the patient] might say that today I would like to watch TV a bit longer… for example.’*  [Healthcare provider, Nilsen 2016]  *‘it should be person-centred, and technology isn’t person-centred… you’ve got the technology, but you can’t use it until he’s capable of accepting [it]… you can’t treat everybody the same, and that’s where technology falls down, because it’d be too [expensive] to personalise it, and then who’d pay for that?’*  [Nurse, Hall 2019]  *‘…these technologies could like bring on a sense of paranoia or bring on some behavioural and psychological symptoms of dementia for someone…oftentimes*  *people talk about being watched, and we brush that off as being a sign of dementia. When in this case…It would be accurate.’*  [KI 12, Direct care provider, Warner, 2023]  *‘I often explain it that we used to, when we were going out for like IndependencePlus and other technology projects in the past, we used to think what technology’s out there and let’s go and buy it and now let’s look at people that we provide care for and fit them to that technology. That is probably the biggest learning, that that’s a mistake. We shouldn’t be doing that.’*  [CS4 P01, Gasby, 2023] |
|  | Concerns about privacy and data security | ‘… there are ethical problems if it’s used as a means to monitor the person…’  [Professional caregiver, Verloo 2020]  *‘I have no problem displaying what I do at work. I rather think of the user, of … Where did the privacy go? I enter and leave the room and do my job, and am supposed to be professional. But the users shall feel that they have a private life when they enter their flat, that they are not going to be under surveillance, 'cause that is unnatural.’*  [Healthcare provider, Nilsen 2016]  *‘…we get lots of issues or questions from folks around privacy… ‘*  [Practitioner, Ding, 2023]  *‘… going back to the whole privacy thing as well, that’s very important to clients. So educating them about how this technology works…’*  [Practitioner, Ding, 2023]  *‘The privacy and confidentiality issue will be very important for the patient, especially when we’re talking about clouds and everything and how to protect their privacy.’*  [Participant 6, Pharmacist, Islam, 2022]  *‘The challenge with this, even as a concept, is this idea of it’s all a bit Big Brother-like, it’s all a bit you know, sort of a bit ‘spying’… The challenge would be to break down some of the stigma that might come with that. I’m not necessarily saying that it’s true, that it’s like a bit Big Brother-like, but I think that is the perception amongst some people who might be resistant to using the technology, you know. If it’s just there forever, recording how many times I use the toilet, you know, it’s uncomfortable.’*  [CS2 P03, Glasby, 2023] |
